# The effect of anastrozole on the pharmacokinetics of tamoxifen in post-menopausal women with early breast cancer

**DOI:** 10.1038/sj.bjc.6690050

**Published:** 1999-01

**Authors:** M Dowsett, J S Tobias, A Howell, G M Blackman, H Welch, N King, R Ponzone, M von Euler, M Baum

**Affiliations:** Academic Department of Biochemistry, Royal Marsden Hospital, London, SW3 6JJ, UK; Meyerstein Institute, Middlesex Hospital, London, W1N 8AA, UK; CRC Department of Medical Oncology, Christie Hospital NHS Trust, Wilmslow Road, Manchester, M20 9BX, UK; Zeneca Pharmaceuticals, Mereside, Macclesfield, Cheshire, SK10 4TG, UK; Institute of Surgical Studies, Charles Bell House, London, W1P 7LD, UK

**Keywords:** Arimidex, tamoxifen, aromatase inhibitor, oestrogen, oestradiol, breast cancer

## Abstract

Thirty-four post-menopausal women with early breast cancer who had received 20 mg tamoxifen once daily as adjuvant therapy for at least 10 weeks participated in a randomized, double-blind, parallel-group, multicentre trial. The primary aim of the trial was to determine the effect of anastrozole upon tamoxifen pharmacokinetics, with secondary aims of assessing the tolerability of the two drugs in combination and whether or not tamoxifen had any effect upon the oestradiol suppression seen with anastrozole. Patients were randomized to receive either 1 mg anastrozole (16 patients) or matching placebo (18 patients) once daily on a double-blind basis for 28 days. No significant difference (*P* = 0.919) was observed in serum tamoxifen concentrations between the anastrozole and placebo groups during the trial. The serum concentration of oestradiol was significantly suppressed (*P* < 0.0001) in patients co-administered anastrozole compared with placebo in the presence of tamoxifen, confirming that anastrozole remained an effective suppressant of oestradiol in the presence of tamoxifen. The combination of tamoxifen and anastrozole was well tolerated, with very little difference in side-effects reported between anastrozole and placebo. In conclusion, the results of this study confirm that anastrozole does not affect the pharmacokinetics of tamoxifen when the two drugs are given in combination to post-menopausal women with early breast cancer. In addition, the oestradiol suppressant effects of anastrozole appear unaffected by tamoxifen. © 1999 Cancer Research Campaign


*British Journal of Cancer* (1999) **79**(2), 311–315
© 1999 Cancer Research Campaign

### The effect of anastrozole on the pharmacokinetics of tamoxifen in post-menopausal women with early breast cancer

**M Dowsett[1], JS Tobias[2], A Howell[3], GM Blackman[2], H Welch[1], N King[1], R Ponzone[1], M von Euler[4] and M Baum[5]**

[1]Academic Department of Biochemistry, Royal Marsden Hospital, London SW3 6JJ, UK; [2]Meyerstein Institute, Middlesex Hospital, London W1N 8AA, UK; [3]CRC Department of Medical Oncology, Christie Hospital NHS Trust, Wilmslow Road, Manchester M20 9BX, UK; [4]Zeneca Pharmaceuticals, Mereside, Alderley Park, Macclesfield, Cheshire SK10 4TG, UK; [5]Institute of Surgical Studies, Charles Bell House, London W1P 7LD, UK

**Summary** Thirty-four post-menopausal women with early breast cancer who had received 20 mg tamoxifen once daily as adjuvant therapy for at least 10 weeks participated in a randomized, double-blind, parallel-group, multicentre trial. The primary aim of the trial was to determine the effect of anastrozole upon tamoxifen pharmacokinetics, with secondary aims of assessing the tolerability of the two drugs in combination and whether or not tamoxifen had any effect upon the oestradiol suppression seen with anastrozole. Patients were randomized to receive either 1 mg anastrozole (16 patients) or matching placebo (18 patients) once daily on a double-blind basis for 28 days. No significant difference ($P = 0.919$) was observed in serum tamoxifen concentrations between the anastrozole and placebo groups during the trial. The serum concentration of oestradiol was significantly suppressed ($P < 0.0001$) in patients co-administered anastrozole compared with placebo in the presence of tamoxifen, confirming that anastrozole remained an effective suppressant of oestradiol in the presence of tamoxifen. The combination of tamoxifen and anastrozole was well tolerated, with very little difference in side-effects reported between anastrozole and placebo. In conclusion, the results of this study confirm that anastrozole does not affect the pharmacokinetics of tamoxifen when the two drugs are given in combination to post-menopausal women with early breast cancer. In addition, the oestradiol suppressant effects of anastrozole appear unaffected by tamoxifen.

**Keywords:** Arimidex; tamoxifen; aromatase inhibitor; oestrogen; oestradiol; breast cancer

Breast cancer is the most common malignancy to affect women in North America and Europe. An estimated 5 000 000 women will be diagnosed with the disease in the next decade (Gelber et al, 1993).

A number of endocrine options are available for the treatment of breast cancer (Howell et al, 1996; Howell and Dowsett, 1997); currently, tamoxifen is the most widely used endocrine agent, with the new generation aromatase inhibitors being increasingly used in post-menopausal women (Buzdar et al, 1996, 1997). Tamoxifen acts primarily as an anti-oestrogen, competing with oestrogen at oestrogen-receptor binding sites in tissues such as the breast. Tamoxifen is used for both the treatment of early and advanced disease. Aromatase inhibitors work by inhibiting aromatase, the enzyme which catalyses the conversion of androgens (androstenedione and testosterone) to oestrogens (oestrone and oestradiol) in a process known as aromatization. In post-menopausal women, oestrogens are mostly derived from adrenal androgens through this conversion.

From the different and complementary modes of action of these two approaches, it is possible that using both in combination may provide a more effective method of treating breast cancer in post-menopausal women. A study is currently being carried out in which tamoxifen is compared with the aromatase inhibitor anastrozole (Arimidex), and the combination of tamoxifen plus anastrozole in post-menopausal women with early breast cancer is also being investigated.

Anastrozole is a potent, selective, non-steroidal aromatase inhibitor belonging to the triazole class. Preclinical pharmacological studies show that anastrozole is a potent inhibitor of placental aromatase, and is selective for the aromatase enzyme complex without affecting the other cytochrome P-450 enzymes involved in the synthesis of adrenal glucocorticoid and mineralocorticoid hormones (Dukes et al, 1996). Anastrozole has been shown to be a well-tolerated (Buzdar et al, 1996) and selective aromatase inhibitor in post-menopausal women (Yates et al, 1996). Doses of anastrozole up to 10 mg once daily do not affect cortisol or aldosterone secretion by the adrenal gland (Plourde et al, 1994). At doses of 1 mg and 10 mg once daily, anastrozole leads to potent inhibition of aromatase (96.7% and 98.1%, respectively, expressed as the geometric mean), and suppresses plasma oestrogens to the limit of detection of available assays (Geisler et al, 1996). Plasma levels of oestrone, oestradiol and oestrone sulphate were suppressed by ≥86.5%, ≥83.5% and ≥93.5% irrespective of dose.

Anastrozole has been compared with megestrol acetate in large randomized trials in post-menopausal women with advanced breast cancer (Buzdar et al, 1996). A recent survival update based on the combined data from these studies (31-month follow-up) showed that treatment with 1 mg anastrozole results in significantly longer survival compared with 160 mg megestrol acetate (Buzdar et al, 1997).

At present, the anti-oestrogen tamoxifen is the endocrine agent of choice as adjuvant therapy for breast cancer in post-menopausal

*Received 19 January 1998*
*Revised 2 June 1998*
*Accepted 9 June 1998*

*Correspondence to:* M Dowsett, Academic Department of Biochemistry, Royal Marsden Hospital, London SW3 6JJ, UK

women. To investigate whether or not the use of tamoxifen in combination with anastrozole provides more effective drug therapy for post-menopausal women with early breast cancer, a large clinical trial has been undertaken. Before initiating this large clinical trial programme comparing anastrozole, tamoxifen and the combination of the two drugs in post-menopausal women with early breast cancer, it was necessary to determine whether anastrozole had any effect on tamoxifen blood levels, and hence on the clinical efficacy of tamoxifen. It is noteworthy that the prototype aromatase inhibitor aminoglutethimide led to an approximate halving of plasma levels of tamoxifen (Lien et al, 1990).

A pharmacokinetic trial was, therefore, carried out to assess the following end points: (1) the effect of anastrozole on the pharmacokinetics of tamoxifen; (2) the safety and tolerability of the combination of anastrozole and tamoxifen therapy; (3) the effect of tamoxifen on the action of anastrozole, with respect to oestradiol suppression. This paper reports the findings of this trial.

### PATIENTS, MATERIALS AND METHODS

### Patient demography

Thirty-four post-menopausal women with early breast cancer who had received 20 mg tamoxifen once daily as adjuvant therapy for at least 10 weeks were recruited from three centres in the UK. Post-menopausal women were defined as those aged 50 years or over who had not menstruated in the last 12 months, or women of any age with follicle stimulating hormone (FSH) levels greater than 40 IU $l^{-1}$. One patient was removed from the study because she was found to be premenopausal.

Exclusion criteria included: premenopausal women or drug-induced menopause; levels of aspartate aminotransferase, alanine aminotransferase, or total bilirubin more than three times the upper limit of the reference range; other abnormal laboratory test results at baseline that would place the patient at risk or confound the results of the trial; and concurrent therapy with a non-approved or experimental drug, or with drugs that might affect steroid hormone status and/or tamoxifen steady-state levels.

The demographic characteristics of all randomized patients are summarized in Table 1. Chemotherapy had previously been given to one patient in the anastrozole group, and six patients in the placebo group. Thirteen patients in the anastrozole group, and 14 patients in the placebo group had previously received radiotherapy. At baseline, the two groups were balanced with respect to demographic variables.

The trial was designed and monitored to comply with the ethical principles of Good Clinical Practice (GCP), and in accordance with the Declaration of Helsinki. All patients included in the trial had given their written informed consent.

**Table 1** Patient demographics

| Demographic characteristics | Anastrozole (1 mg) | Placebo |
|---|---|---|
| Number of patients exposed | 16 | 18 |
| Age (years) | | |
| *n* | 16 | 18 |
| Median | 64.0 | 61.0 |
| Minimum | 51 | 42 |
| Maximum | 81 | 73 |
| Height (cm) | | |
| *n* | 15 | 18 |
| Median | 162.0 | 165.0 |
| Minimum | 152.0 | 150.0 |
| Maximum | 178.0 | 172.0 |
| Weight (kg) | | |
| *n* | 15 | 18 |
| Median | 73.0 | 71.5 |
| Minimum | 56.9 | 53.3 |
| Maximum | 92.1 | 100.8 |
| Origin (number of patients) | | |
| Caucasian | 16 | 17 |
| Afro-Caribbean | 0 | 1 |

The height and weight of one patient in the anastrozole group were not taken.

### Study design

The trial was a randomized, double-blind, parallel-group, multi-centre trial. All patients had been taking 20 mg tamoxifen for at least 10 weeks. All patients continued to take 20 mg tamoxifen orally once a day throughout the trial, and, in addition, were randomized to receive either 1 mg anastrozole (16 patients) or matching placebo (18 patients) once daily on a double-blind basis. Randomized treatment started on day 0 and ended on day 28. To assess any post-treatment effects of the combination therapy of anastrozole and tamoxifen, all patients were required to continue taking tamoxifen alone for another 14 days (day 42).

Blood samples were taken from each patient at days –7 and 0 for baseline measurements; days 14 and 28 for on-treatment measurements, and day 42 for post-treatment measurements. Tamoxifen and anastrozole/placebo tablets were taken together at the same time each day so that blood samples could be taken at trough drug levels.

### Key assessments

Serum levels of tamoxifen were analysed according to the high-performance liquid chromatography method described by Johnston et al (1993). Overall, recovery varied from 79% to 93% and was corrected for by the internal recovery control. The intra-assay coefficient of variation was 2.2% at a mean level of 135 ng $ml^{-1}$. The sensitivity limit was <5 ng $ml^{-1}$. Plasma levels of anastrozole were determined using a validated method involving solvent extraction, capillary gas chromatography, and separation with electron capture detection (ECD).

Serum levels of oestradiol were determined using a method previously reported (Dowsett et al, 1987). Because of variability of baseline oestradiol concentrations among individuals, oestradiol values were expressed as the oestradiol proportion, defined as the geometric mean of the levels of oestradiol measured during treatment (days 14 and 28) divided by the geometric mean of the levels of oestradiol measured at baseline (days –7 and 0). Any oestradiol values that lay below the limit of quantification were substituted by the value assigned as the limit of quantification (3 pmol $l^{-1}$).

### Tolerability and safety assessments

An adverse event was defined as any detrimental change in a patient's condition during the study. All adverse events were

 © Cancer Research Campaign 1999

**Table 2** Serum tamoxifen concentrations during co-administration of 20 mg tamoxifen and either 1 mg anastrozole or placebo

| | **Tamoxifen concentration (ng ml$^{-1}$)** | | | | |
|---|---|---|---|---|---|
| **Day** | **–7** | **0** | **14** | **28** | **42** |
| Anastrozole group | | | | | |
| *n* | 15 | 15 | 15 | 15 | 15 |
| Mean | 171.0 | 154.8 | 156.8 | 153.1 | 160.6 |
| Standard deviation | 76.8 | 71.7 | 66.0 | 72.9 | 73.1 |
| Placebo group | | | | | |
| *n* | 18 | 18 | 18 | 18 | 18 |
| Mean | 123.8 | 116.4 | 121.7 | 117.1 | 123.4 |
| Standard deviation | 54.9 | 46.4 | 40.3 | 45.0 | 50.3 |

One patient was removed from the study because she was found to be premenopausal.

recorded, irrespective of whether or not the event was considered to be related to the trial therapy. Any clinically significant abnormal laboratory results recorded at any time were reported as adverse events. Biochemical and haematological variables were monitored throughout the trial to assess drug safety.

### Statistical analyses

The primary end points of this trial were serum tamoxifen levels and oestradiol proportion, with the treatment comparison of interest being that of anastrozole with placebo.

The comparison of the serum tamoxifen levels between the two groups was performed by analysis of covariance (ANCOVA). The two groups were compared with respect to the 'on-treatment' tamoxifen level. The 'on-treatment' value was derived by taking the geometric mean of the day 14 and day 28 tamoxifen concentrations. The estimate of the difference in 'on-treatment' tamoxifen levels between the two groups was derived by taking the difference in the least square mean values obtained from the ANCOVA.

The comparison of the oestradiol levels was performed in a similar way to the analysis of tamoxifen levels, the methods differed only by the derivation of the end point for analysis. The analysis of oestradiol levels considered the 'oestradiol proportion'. The 'oestradiol proportion' was defined as the 'on treatment' oestradiol level divided by the 'baseline' oestradiol level, with 'on treatment' and 'baseline' defined as above.

### RESULTS

### Serum concentrations of tamoxifen

The serum tamoxifen concentrations (means±standard deviation) during the trial are summarized in Table 2. As the data followed a normal distribution, relevant arithmetic descriptive statistics are given. In both patient groups, the steady-state tamoxifen concentrations remained similar both before and during co-administration of 20 mg tamoxifen and either 1 mg anastrozole or placebo. A difference was seen in the baseline levels of tamoxifen between the anastrozole and placebo groups. However, when the data were adjusted for baseline, there was no significant difference in tamoxifen concentrations between these two groups during treatment ($P$ = 0.919) (Figure 1). There was no significant difference ($P$ = 0.953) in tamoxifen concentrations between the anastrozole and placebo groups during the post-treatment period, as measured on day 42 (Figure 1). Therefore, co-administration of 1 mg anastrozole and 20 mg tamoxifen did not significantly affect the steady-state serum concentrations of tamoxifen.

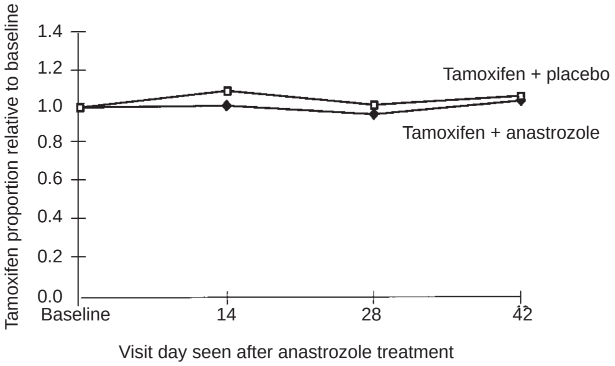

**Figure 1** The mean tamoxifen proportion relative to baseline geometric mean by treatment group

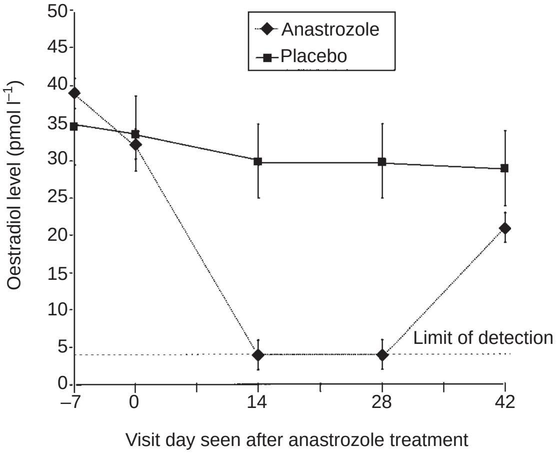

**Figure 2** Mean oestradiol serum concentration (±s.e.) during treatment for 28 days with 20 mg tamoxifen and either 1 mg anastrozole daily or placebo

### Serum concentrations of oestradiol

In patients taking placebo in the presence of tamoxifen, the serum oestradiol concentrations on days 14 and 28 were similar to baseline concentrations (days –7 and 0) and post-treatment (day 42) concentrations when tamoxifen was taken alone (Figure 2). The serum concentrations of oestradiol on days 14 and 28 in patients taking anastrozole in the presence of tamoxifen were markedly decreased compared with baseline concentrations. There was a significant difference ($P$ < 0.0001) for the on-trial treatment oestradiol proportion, adjusted for baseline, between the anastrozole and placebo groups.

### Plasma concentrations of anastrozole

The mean plasma concentrations of anastrozole measured on days 14 and 28, when patients were taking the combination of tamoxifen and anastrozole, were 33.2±10.2 ng ml$^{-1}$ and 33.9 ± 12.0 ng ml$^{-1}$ respectively.

© Cancer Research Campaign 1999 

### Tolerability and safety evaluation

A total of 18 patients (52.9%) experienced adverse events during the trial. In the anastrozole group, nine patients experienced a total of 25 adverse events whereas, in the placebo group, nine patients experienced a total of 28 adverse events. Overall, the incidence of adverse events in the two groups was similar, 56.3% of patients treated with anastrozole (1 mg) and 50% of patients treated with placebo reporting at least one adverse event.

Hot flushes were experienced by four patients, all of whom were taking tamoxifen and placebo. Digestive disorders comprising diarrhoea, nausea and vomiting were experienced by five patients in the anastrozole group and one patient in the placebo group. Of the five patients in the anastrozole group, two patients experienced diarrhoea, two patients experienced nausea, and one patient experienced vomiting. The patient in the placebo group experienced nausea.

There was one withdrawal due to adverse events (from the placebo group) and one serious adverse event (dental pain, which occurred in the anastrozole group); the latter was reported before treatment commenced. No deaths were reported during the study.

No apparent differences were seen between either treatment group or within each group with respect to pre- and post-treatment haematology and biochemistry measurements.

### DISCUSSION

Before initiating the adjuvant breast cancer trial comparing anastrozole with tamoxifen and the combination of anastrozole plus tamoxifen in post-menopausal women, it was important to investigate whether the combination of the two drugs had any effect upon plasma tamoxifen levels because any reduction in tamoxifen could, in theory, affect the clinical efficacy of tamoxifen. This study was appropriate because in earlier studies of the prototype aromatase inhibitor aminoglutethimide, in combination with tamoxifen, aminoglutethimide showed an interaction with tamoxifen which led to a 50% reduction in plasma levels of the anti-oestrogen (Lien et al, 1990). The combination of aminoglutethimide and tamoxifen has been compared clinically with tamoxifen alone in post-menopausal women with advanced breast cancer (Milstead et al, 1985; Ingle et al, 1986; Rose et al, 1986; Smith et al, 1986); in none of these studies was there a significant enhancement of efficacy with the combination. It may be speculated that this might be due in part to the lower tamoxifen levels with combined therapy. Tamoxifen is extensively metabolized by cytochrome P-450-dependent hepatic mixed function oxidase (Jacolot et al, 1991) and aminoglutethimide is a powerful cytochrome P-450 inducer.

Anastrozole is more selective than aminoglutethimide, and a weak inducer of cytochrome P-450, with patterns of induced enzymes dissimilar to aminoglutethimide. Co-administration of anastrozole with a variety of compounds, for example antipyrine, cimetidine and warfarin, indicate that anastrozole is unlikely to result in clinically significant drug interactions mediated by cytochrome P-450 (Dowsett et al, 1998). Nonetheless, in vivo pharmacokinetic interactions are not always predictable. Thus, a pilot clinical study before the large adjuvant trial was considered prudent.

The results of this study confirmed that serum tamoxifen concentrations were unaffected by the presence of anastrozole, as shown by the unchanged serum tamoxifen concentrations compared both with the placebo group and with the post-treatment period in which tamoxifen alone was given to all patients. A difference in baseline serum concentrations of tamoxifen was observed between the anastrozole and placebo groups. This is consistent with the considerable inter-patient variability in tamoxifen concentrations previously reported in studies involving post-menopausal women with advanced breast cancer (Patterson et al, 1980; Bratherton et al, 1984; Frenay et al, 1994). The difference in baseline serum concentrations of tamoxifen is very unlikely to have affected the results of the study, because the longitudinal design with a parallel placebo group allowed the on-treatment values to be adjusted for baseline.

The results of this trial also confirmed that oestradiol concentrations were suppressed in patients given a combination of anastrozole plus tamoxifen for a period of 28 days. The levels of suppression seen in this study were highly comparable to those seen in a study in which anastrozole was taken alone (Geisler et al, 1996). Because there was not an anastrozole alone arm or treatment period in this current trial, it is not possible to exclude a minor effect of tamoxifen on the action of anastrozole; however, these data clearly demonstrate that anastrozole remains a very effective suppressant of oestradiol in the presence of tamoxifen.

The plasma levels of anastrozole obtained in this trial of patients with early breast cancer were informally compared with those from two other clinical trials in which advanced breast cancer patients received 1 mg anastrozole once daily for 60 weeks, but in which anastrozole levels were taken at day 28 ($n$ = 95) (Zeneca, data on file) or for 28 days ($n$ = 10) (Geisler et al, 1996). Steady-state concentrations of anastrozole in patients taking 1 mg anastrozole in these trials ranged from approximately 9.0 to 108.0 ng ml$^{-1}$ and from 22.0 to 83.9 ng ml$^{-1}$, respectively, and in the present trial from 22.0 to 60.0 ng ml$^{-1}$ on day 14 and 14.0 to 66.0 ng ml$^{-1}$ on day 28. The mean concentration of anastrozole was 39.0±14.7 ng ml$^{-1}$ in the 60-week trial ($n$ = 95), 37.7 ng ml$^{-1}$ in the 28-day trial ($n$ = 10), and 33.2±10.2 ng ml$^{-1}$ on day 14 and 33.9±12.0 ng ml$^{-1}$ on day 28 of the combination trial. Although definitive conclusions as to whether or not tamoxifen affects anastrozole blood levels cannot be made, studies are in progress specifically to examine this question. It is important to note, however, that if such an interaction exists between tamoxifen and anastrozole, it is clearly insufficient to reduce the oestrogen-suppressive effects of anastrozole.

The combination of tamoxifen plus anastrozole treatment was well tolerated during the study. No unexpected adverse events were related to the use of combination therapy, and no significant difference in adverse events was reported between the anastrozole and placebo groups. These data are supportive of the use of the combination of tamoxifen plus anastrozole in the long-term adjuvant setting.

In conclusion, the results of the present study confirm that anastrozole did not affect the pharmacokinetics of tamoxifen when the two drugs were given in combination to post-menopausal women with early breast cancer. Thus, in the on-going adjuvant trial, one can be assured that for patients randomized to combined therapy, full-dose tamoxifen will be received. In addition, the oestradiol suppressant effects of anastrozole appear to be unaffected by tamoxifen. If tamoxifen has any effect on anastrozole blood levels, such an interaction is clearly insufficient to reduce the oestradiol suppressive effects of anastrozole.

### REFERENCES

Bratherton DG, Brown CH, Buchanan R, Hall V, Kingsley Pillers EM, Wheeler TK and Williams CJ (1984) A comparison of two doses of tamoxifen (Nolvadex) in postmenopausal women with advanced breast cancer: 10 mg bd versus 20 mg bd. *Br J Cancer* **50**: 199–205

© Cancer Research Campaign 1999

Buzdar A, Jonat W, Howell A, Jones SE, Blomqvist C, Vogel CL, Eirmann W, Wolter JM, Azab M, Webster A and Plourde PV for the Arimidex Study Group (1996) Anastrozole, a potent and selective aromatase inhibitor, versus megestrol acetate in postmenopausal women with advanced breast cancer: result of overview analysis of two phase III trials. *J Clin Oncol* **14**: 2000–2011

Buzdar A, Jonat W, Howell A, Yin H and Lee D on behalf of the Arimidex International Study Group (1997) Significant improved survival with Arimidex (anastrozole) versus megestrol acetate in postmenopausal advanced breast cancer: updated results of two randomized trials (abstract 545). *Proc Am Soc Clin Oncol* **16**: 156a

Dowsett M, Goss PE, Powles TJ, Hutchinson G, Brodie AMH, Jeffcoate SL and Coombes RC (1987) Use of the aromatase inhibitor 4-hydroxyandrostenedione in postmenopausal breast cancer: optimization of therapeutic dose and route. *Cancer Res* **47**: 1957–1961

Dowsett M, Yates R and Wong WJ (1998) 'Arimidex' (anastrozole): lack of interactions with tamoxifen, antipyrine, cimetidine and warfarin. *Eur J Cancer* **34** (suppl. 1): S39–S40

Dukes M, Edwards PN, Large M, Smith IK and Boyle T (1996) The preclinical pharmacology of 'Arimidex' (Anastrozole; ZD1033) – a potent selective aromatase inhibitor. *J Steroid Biochem Mol Biol* **58**: 439–445

Frenay M, Peyrade F, Étienne MC, Ruch F, Milano G, François E, Ferrero JM, Ibrahim-Gobert C and Namer M (1994) Population pharmacokinetics of tamoxifen and its metabolites: a study of 316 patients with breast cancer (abstract). *Bulletin du Cancer* **81**: 489–490

Geisler J, King N, Dowsett M, Ottestad L, Lundgren S, Walton P, Kormeset PO and Lønning PE (1996) Influence of anastrozole (Arimidex), a selective, non-steroidal aromatase inhibitor, on in vivo aromatization and plasma oestrogen levels in post-menopausal women with breast cancer. *Br J Cancer* **74**: 1286–1291

Gelber R, Goldhirsch A and Coates AS (1993) Adjuvant therapy for breast cancer. Understanding the overview. *J Clin Oncol* **11**: 580–585

Howell A and Dowsett M (1997) Recent advances in endocrine therapy of breast cancer. *Br Med J* **315**: 863–866

Howell A, Downey S and Anderson E (1996) New endocrine therapies for breast cancer. *Eur J Cancer* **32A**: 576–588

Ingle JN, Green SJ, Ahmann D, Long HJ, Edmonson JH, Rubin J, Chang MN and Creagan ET (1986) Randomized trial of tamoxifen alone or combined with aminoglutethimide and hydrocortisone in women with metastatic breast cancer. *J Clin Oncol* **4**: 958–964

Jacolot F, Simon I, Dreano Y, Beaune P, Riche C and Berthou F (1991) Identification of the cytochrome P450 IIIA family as the enzymes involved in the *N*-demethylation of tamoxifen in human liver microsomes. *Biochem Pharmacol* **41**: 1911–1919

Johnston SRD, Haynes BP, Sacks NPM, McKinna JA, Griggs LJ, Jarman M, Baum M, Smith IE and Dowsett M (1993) Effect of oestrogen receptor status and time on the intratumoural accumulation of tamoxifen and *N*-desmethyltamoxifen following short-term therapy in human breast cancer. *Breast Cancer Res Treat* **28**: 241–250

Lien EA, Anker G, Lønning PE, Solheim E and Ueland PM (1990) Decreased serum concentrations of tamoxifen and its metabolites induced by aminoglutethimide. *Cancer Res* **50**: 5851–5857

Milstead R, Habeshaw T, Kaye S, Sangster G, Macbeth F, Campbell-Ferguson J, Smith D and Calman K (1985) A randomized trial of tamoxifen versus tamoxifen and aminoglutethimide in postmenopausal women with advanced breast cancer. *Cancer Chemother Pharmacol* **14**: 272–273

Patterson JS, Settatree RS, Adam HK and Kemp JV (1980) Serum concentrations of tamoxifen and major metabolite during long term Nolvadex therapy, correlated with clinical response. *Eur J Cancer* (suppl. 1): 89–92

Plourde PV, Dyroff M and Dukes M (1994) Arimidex: a potent and selective fourth-generation aromatase inhibitor. *Breast Cancer Res Treat* **30**: 103–111

Rose C, Kamby C, Mourisden HT, Bastholt L, Brincker H, Skovgaard-Poulsen H, Andersen AP, Loft H, Dombernowsky P and Andersen KW (1986) Combined endocrine treatment of postmenopausal patients with advanced breast cancer. A randomized trial of tamoxifen vs. tamoxifen plus aminoglutethimide and hydrocortisone. *Breast Cancer Res Treat* **7** (suppl.): S45–50

Smith IE, Macaulay V and Bozek T (1986) Comparative studies of 'Nolvadex' and aminoglutethimide alone and in combination in advanced breast cancer. *Rev Endocr Related Cancer* (suppl. 18): 21–25

Yates RA, Dowsett M, Fisher GV, Selen A and Wyld PJ (1996) Arimidex (ZD1033): a selective, potent inhibitor of aromatase in postmenopausal female volunteers. *Br J Cancer* **73**: 543–548

*© Cancer Research Campaign 1999*
